# Equity Meets Primary-Care-Sensitive Performance: A Longitudinal Benchmark of Cost-Related Unmet Care and Avoidable Hospitalization in Europe

**DOI:** 10.3390/healthcare14193294

**Published:** 2026-10-02

**Authors:** Fatih Özcan, Tuna Can Güleç, Merve Vatansever Balcan, Elif Sıdal Gültekin, Sena Sönmez

**Affiliations:** 1 Department of Family Medicine, Medical Faculty of Manisa Celal Bayar University, Manisa 45030, Türkiye; merve.balcan@cbu.edu.tr (M.V.B.); elif.gultekin@cbu.edu.tr (E.S.G.); sena.sonmez@cbu.edu.tr (S.S.); 2 Department of Capital Markets, Faculty of Applied Sciences, Manisa Celal Bayar University, Manisa 45140, Türkiye; tunacan.gulec@cbu.edu.tr

**Keywords:** health-system performance, health inequality, unmet medical need, primary care, avoidable hospitalization, health expenditure

## Abstract

Background/Objectives: This study compares European health systems by considering the cost-related unmet-care inequality between income groups and primary-care-sensitive avoidable hospitalizations. Methods: This was a longitudinal comparative benchmarking study. The main sample consists of 275 country-year observations from 20 European countries with at least ten years of data during 2009–2023. The first dimension is measured by the absolute difference between the lowest and highest income quintiles in the prevalence of unmet medical examination or treatment because it was too expensive. The second dimension uses age- and sex-standardized avoidable hospitalization rates for asthma, chronic obstructive pulmonary disease, congestive heart failure, and diabetes. The 20-country core sample was defined before within-year rank normalization. The indicators were converted into rank scores on a scale of 0–1 within each annual comparison set, with equal weighting for both dimensions. Temporal uncertainty in country rankings was examined using a three-year synchronized moving-block bootstrap with 10,000 iterations. Results: In the main comparison, the Netherlands, Slovenia, Spain, Sweden, and Finland ranked in the top five. The Netherlands and Slovenia remained in the top five in all valid bootstrap replications; the corresponding probabilities were 99.90% for Spain, 90.67% for Sweden, and 95.66% for Finland. Top-five membership nevertheless changed under some alternative weighting, disease-omission, scaling, and balanced-period specifications. In the spending-adjusted assessment, each country was compared based on a nonlinear spending–performance relationship established from other countries, without including its own observations in the estimation equation; Slovenia, the Netherlands, and Spain took the top three positions. Conclusions: The findings should be interpreted not as a definitive country ranking, but as a relative assessment of health systems that highlights the differences between health inequality, primary care-sensitive outcomes, and resource intensity.

## 1. Introduction

The success of health systems cannot be explained solely by individual indicators such as life expectancy, mortality rate, or per capita health expenditure. A health system must simultaneously respond to the health needs of the population, maintain the quality of care, limit inequalities between social groups, and transform available resources into reasonable outcomes. The approach that considers health system performance within this multidimensional framework is not new; the early frameworks of the World Health Organization and later international comparative studies have long emphasized the importance of the distinction between efficiency, equity, quality, and resource use [1,2,3]. The OECD’s updated health-system performance assessment framework also defines equity and efficiency as cross-cutting dimensions of health-system performance [4].

One of the most visible areas of this multidimensionality is social disparities in access to healthcare. It has been shown that the concept of access cannot be explained solely by the supply side; it is a multi-stage process extending from an individual’s perception of their need to accessing and benefiting from the service [5]. The persistence of health disparities in Europe based on income, education, and other socioeconomic characteristics also indicates that national averages can mask distributional problems [6,7]. The included countries also represent heterogeneous health-system arrangements, with differences in financing, cost sharing, gatekeeping, decentralization, and primary-care organization; these institutional differences are treated as contextual heterogeneity rather than causal explanatory variables in the present benchmark [4,8]. A brief country-level summary of financing and primary-care arrangements is provided in Supplementary Table S6 [9].

The European Union Statistics on Income and Living Conditions (EU-SILC) is one of the main comparative data sources that allows monitoring of unmet medical needs across countries and income groups. Previous studies have shown that the likelihood of foregoing or having unmet medical needs increases as income levels decrease; this disparity can become more pronounced during periods of economic contraction [10,11]. The traditionally published rate uses the entire valid sample as the denominator, rather than only those reporting a healthcare need. Therefore, the indicator reflects both the probability of a healthcare need arising and the probability of that need not being met [12]. This study therefore considers this variable not as “access probability”, but as the difference in the prevalence of unmet medical needs due to cost among income groups. Asthma, chronic obstructive pulmonary disease (COPD), congestive heart failure (CHF), and diabetes are diseases where the need for hospitalization can be reduced through regular monitoring, continuation of medication, support for patient self-management, and early detection of exacerbations. The OECD considers these four hospitalization indicators to be among those that could be reduced with better management at the primary care level [13,14,15]. Systematic reviews also reveal a general inverse relationship between access to primary care, physician supply and continuity of care, and hospitalizations for ambulatory-care-sensitive conditions, but these indicators do not solely reflect the quality of primary care [16,17,18].

Therefore, instead of merging indicators under a single broad heading in health system assessments, it is necessary to maintain conceptually separate dimensions and clearly state aggregation decisions [8,19].

The normalization method, weight selection, whether or not inter-indicator compensation is allowed, the number of countries varying by year, and measurement errors can alter country rankings. Current international health system comparisons also show a tendency to present rankings not in a single, definitive order, but with consideration for uncertainty and methodological sensitivity [20]. Recently, studies ranking OECD countries using multi-criteria decision-making methods have also increased the diversity of methods in the field of health system performance [21]. Therefore, the originality of this study is not in “ranking countries”; it is in bringing together income-related unmet-care inequality and primary-care-sensitive avoidable admissions within a single, long-term, uncertainty-aware, and spending-adjusted framework. The study has four objectives: first, to determine the joint relative positions of European health systems in terms of cost-related unmet-care inequality and primary-care-sensitive avoidable admissions; second, to examine the extent to which these positions are sensitive to different weighting, aggregation, period, disease, and scaling choices; third, to measure the temporal uncertainty in country rankings using a moving-block bootstrap that does not rely on the assumption of independent years; and fourth, to compare countries based on the performance level predicted by health spending, without including their own observations in the spending curve.

## 2. Materials and Methods

### 2.1. Data Sources and Sample

Only publicly available, aggregated country-level data were used in the study. Indicators of medical examinations or treatments unaffordable due to cost were taken from Eurostat’s EU-SILC-based hlth_silc_08 dataset [22]. Both sexes, population aged 16 years and over, the “too expensive” reason, and income quintiles were selected. The lowest income quintile was defined as Q1, and the highest as Q5; the basic inequality indicator was calculated as the absolute difference between Q1 and Q5. Primary-care-sensitive admission indicators were taken from the Primary Care–Avoidable Hospital Admissions section of the OECD Health Care Quality and Outcomes database [23]. Age- and sex-standardized rates per 100,000 population were used for asthma (ADMRASTH), COPD (ADMRCOPD), CHF (ADMRCHFL), and diabetes (ADMRDBUC) in the population aged 15 years and over. The OECD diabetes indicator covers hospital admissions with diabetes as the principal diagnosis among persons aged 15 years and older. The age thresholds differ because the two domains follow the standard definitions of their source datasets: EU-SILC reports unmet need for persons aged 16 years and older, whereas the OECD admission indicators refer to persons aged 15 years and older. Avoidable hospitalization was selected as a harmonized, outcome-based measure that is sensitive to primary-care performance; it does not represent the full scope of primary care, which also includes continuity, prevention, coordination, referral practices, and other dimensions.

The health expenditure variable is taken from the SH.XPD.CHEX.PP.CD series published by the World Bank via World Development Indicators. The indicator gives current per capita health expenditure in current international dollars according to purchasing power parity; the primary source is the WHO Global Health Expenditure Database [24].

The joint dataset was established for the period 2009–2023. To be included in the main country comparison, a country had to have at least ten years of joint observations across the two outcome domains. As a result of this rule, 20 countries and 275 country-year observations were included in the main sample. Countries with shorter series were excluded before annual rank normalization; therefore, only the prespecified 20-country core set determined the main annual scores and ranking. The number of observed years per country in the main sample ranged from 10 to 15. The study indicators are summarised in Table 1.

### 2.2. Score Construction

Cost-related unmet-care inequality was measured for each country-year as the absolute difference between Q1 and Q5. In this study, the equity-related dimension is operationalized as |Q1–Q5|; a smaller gap indicates more favorable equity-related performance. This measure captures one distributional aspect of healthcare access and is not intended as a comprehensive measure of health equity. Because Q5 values were close to zero in many countries, Q1/Q5 ratio measures were not used; small denominators could make the measure of relative inequality excessively volatile. Since the units of measurement for the indicators differed, the raw values were not directly aggregated. Each indicator was ranked among the 20 core countries within the same year and converted to a 0–1 range using the following transformation:

*Score_ijt_* = (*N_jt_* − *Rank_ijt_*)/(*N_jt_* − 1) (1)

Here, *N* represents the number of core-sample countries observed for the relevant indicator and year, and Rank indicates the country rank where a lower raw value is considered better. Tied observations were assigned their average rank within the corresponding year. The scores of the four diseases were averaged with equal weights to create a primary-care-sensitive hospitalization domain. The cost-related unmet-care inequality score and the hospitalization score were given equal conceptual weights:

Joint Performance_it_ = 0.50 × Inequality Score_it_ + 0.50 × Hospitalization Score_it_ (2)

Giving equal weight to the two domains is not a data-driven outcome; it is a clear normative choice in the research design. Since four hospitalization indicators would constitute 80% of the total score if all five individual indicators were given equal weight, the domains were equally weighted in the main model. However, 40/60, 60/40, equal weighting of all five indicators, geometric mean, and a non-compensatory criterion based on the lower of the two domains were tested in separate sensitivity analyses. The joint score is a relative summary of these two domains and should not be interpreted as a latent measure of overall health-system quality; a score of 1 indicates the best relative position within the contemporaneous comparator set, not an absolute performance threshold.

### 2.3. Ranking Uncertainty and Sensitivity Analyses

To assess the stability of country rankings over time, a three-year synchronized moving-block bootstrap with 10,000 iterations was applied. Years were selected in blocks of three consecutive years instead of being drawn independently; blocks of the same calendar years were used together across all countries. In the unbalanced main panel, missing country-years were skipped when calculating a country’s resampled mean; a replication was treated as valid for that country when at least one sampled observation was available. The number of valid three-year bootstrap replications ranged from 9995 to 10,000 across countries. The 2.5th percentile was rounded downward and the 97.5th percentile upward to obtain the integer bootstrap rank intervals reported in the table in Section 3.2. Two-year blocks were used as an additional robustness test. The sensitivity of the main country ranking to methodological decisions was examined using Spearman rank correlations. To account for the possibility that differing temporal coverage might affect the rank intervals, the moving-block bootstrap was also repeated in the balanced comparator set consisting of 18 countries with data available for all eight years from 2016 to 2023. In this test, the same 18 countries were compared each year.

### 2.4. Spending-Adjusted Performance

In the second phase of the study, the country’s observed performance was compared with the level predicted by health expenditure. The aim was not to estimate a causal “return to spending,” but to differentiate countries that perform relatively above or below expectations under similar resource intensity conditions. Since the linear model shows substantially weaker fit than nonlinear models, the natural logarithm and square of per capita health expenditure, together with year fixed effects, were used in the baseline calibration:

*Joint Performance_it_* = *β*_1_
*ln*(Spending_i_*_t_*) + *β*_2_ [*ln*(*Spending_it_*)]^2^ + *Year_t_* + *ε_it_* (3)

Each country was excluded from the equation that determined its expected value. In other words, when evaluating country c, the model was built solely on observations from other countries, and the expected performance of country c was estimated from this out-of-country equation. The difference between the actual score and the estimated score is the out-of-country spending-adjusted performance residual. A positive residual means the country’s overall performance is higher than predicted from health expenditure and year conditions; a negative residual means it is lower. This residual was not interpreted as technical efficiency. Since health needs, price level, labour-force composition, disease burden, institutional structure, and other inputs were not fully modelled, the production frontier is not defined [25]. A one-year lagged value of the expenditure measure, a three-year moving average, the share of health expenditure in GDP, a model without year fixed effects, and a flexible spline curve were used as additional tests. For the three-year expenditure sensitivity, the mean was calculated only when expenditure data were available for the current year and both preceding consecutive years; the lags were constructed from the full expenditure series rather than from the outcome-analysis panel. Linear, quadratic, and spline forms were additionally compared using leave-one-country-out root mean squared error (RMSE) and mean absolute error (MAE), alongside AIC. These two leave-one-country-out uses are distinct: the first constructs each country’s spending-adjusted residual, whereas the second uses held-out prediction errors to compare functional forms. The final replication code was verified using Python 3.13.5 (Python Software Foundation, Beaverton, OR, USA) with NumPy 2.3.5 (NumPy Project, NumFOCUS, Inc, Austin, TX, USA), pandas 2.2.3 (pandas development team, NumFOCUS, Inc, Austin, TX, USA) SciPy 1.17.0 (SciPy development team, NumFOCUS, Inc, Austin, TX, USA), statsmodels 0.14.6 (statsmodels development team, NumFOCUS Affiliate, Inc, Austin, TX, USA), Patsy 1.0.2 (Smith, N. J, Berkeley, CA, USA), and Matplotlib 3.10.8. (NumFOCUS, Inc, Austin, TX, USA).

## 3. Results

### 3.1. Descriptive Results

In the main sample, the mean prevalence of cost-related unmet medical need is 2.15% in the lowest income quintile and 0.26% in the highest income quintile. The average absolute difference between the two groups is 1.89 percentage points. The average avoidable hospitalization rate per 100,000 population is 34.5 for asthma, 175.5 for COPD, 268.9 for CHF, and 124.7 for diabetes. The average per capita health expenditure is approximately 4407 current international dollars on a PPP basis. Descriptive statistics are reported in Table 2.

### 3.2. Joint Performance Ranking and Two-Domain Country Profiles

The highest mean joint performance score on the adjusted 0–1 scale was observed in the Netherlands (0.748). The Netherlands was followed by Slovenia (0.730), Spain (0.680), Sweden (0.636), and Finland (0.621). This ranking does not reflect clinical outcomes alone or income inequality alone; it is a weighted joint position of the two domains. The complete country ranking is reported in Table 3.

**Figure 1:**
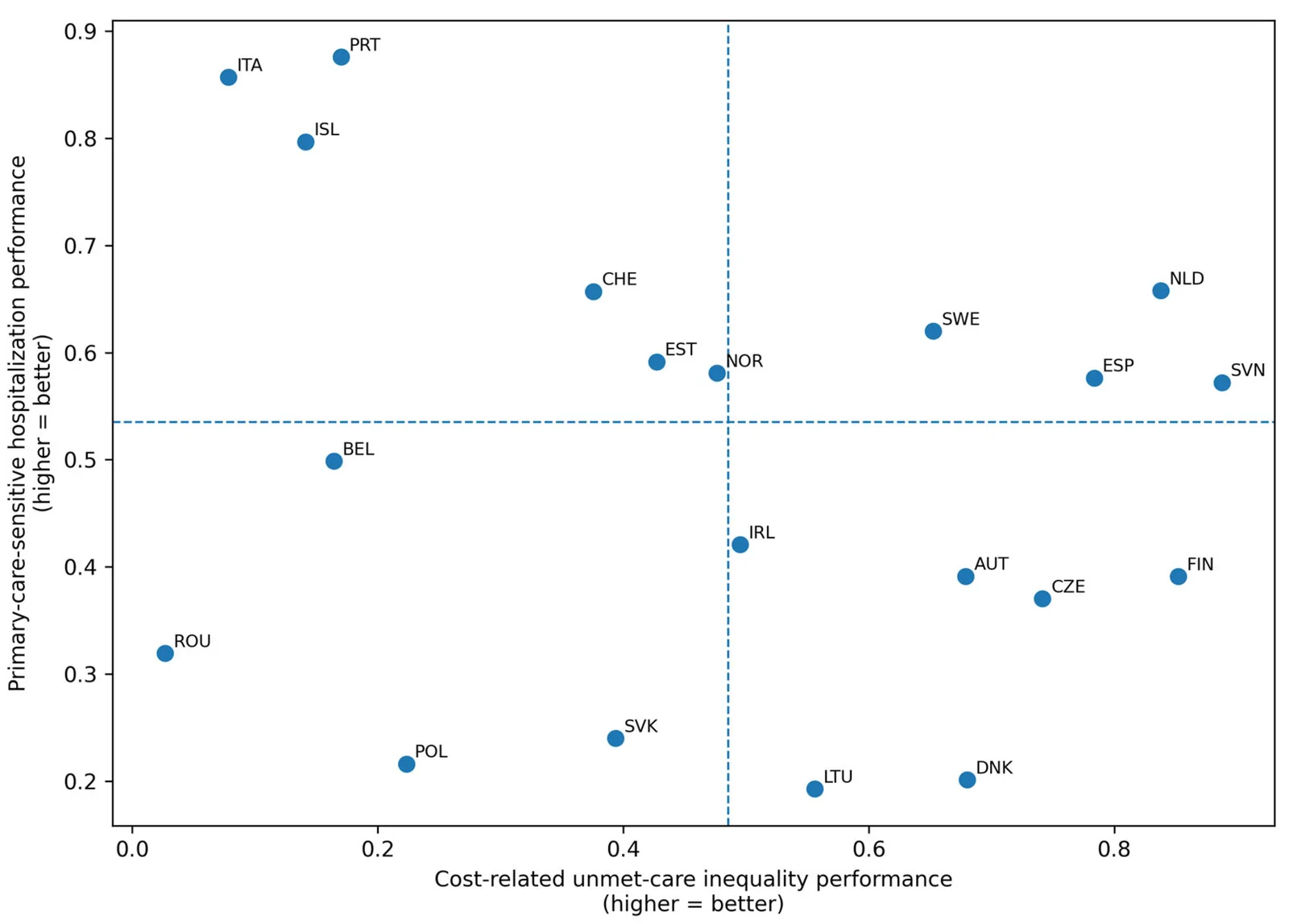
Cost-related unmet-care inequality and primary-care-sensitive hospitalization performance. Note. Dashed lines show the medians of the two domains across the 20 countries. Scores are country averages of relative positions within the annual comparison set, not absolute health levels. Source: Authors’ calculations.

The countries’ positions in the two domains reveal the differentiation behind the joint score. Some countries are relatively strong in both domains; others perform well on cost-related inequality but remain at a moderate level in the hospitalization indicators, or vice versa. Therefore, instead of a joint score, the two domains are shown side-by-side in Figure 1.

### 3.3. Ranking Uncertainty

**Figure 2:**
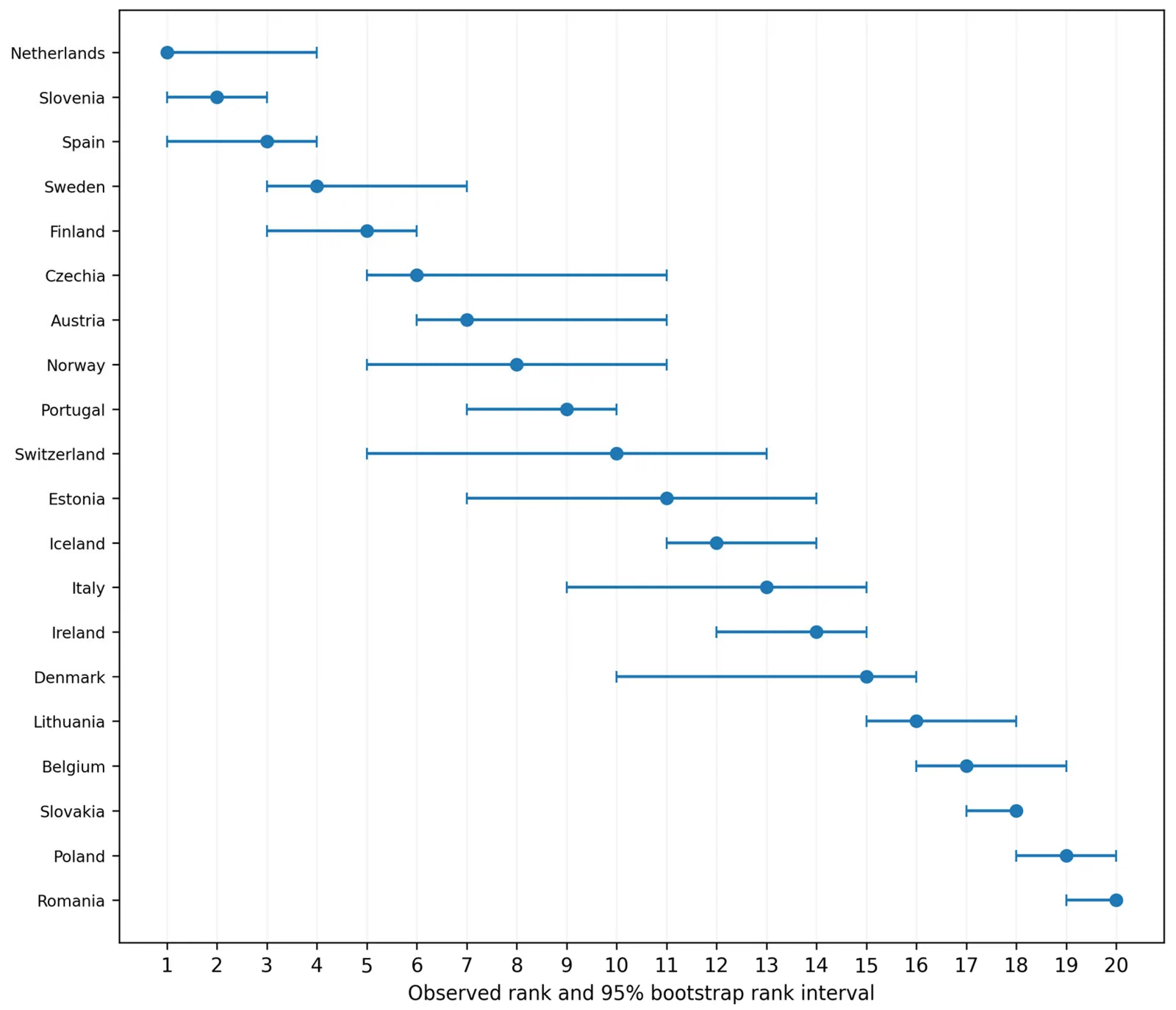
Temporal uncertainty in country rankings. Note. Horizontal lines show the 95% rank interval from the three-year synchronized moving-block bootstrap. Source: Authors’ calculations.

Three-year moving-block bootstrap produced wider and more conservative rank intervals than resampling based on the assumption of independent years. The Netherlands’ 95% bootstrap rank interval is 1–4, Slovenia’s is 1–3, and Spain’s is 1–4. The probabilities of these three countries remaining in the top five are 100%, 100%, and 99.90%, respectively. Sweden and Finland also remain in the upper group, but their rank intervals are 3–7 and 3–6; their top-five probabilities are 90.67% and 95.66%. The two-year block specification produced similar results, with top-five probabilities of 100%, 100%, 99.95%, 95.29%, and 97.19% for the Netherlands, Slovenia, Spain, Sweden, and Finland, respectively. In the balanced 18-country 2016–2023 analysis, Spain, Sweden, the Netherlands, Slovenia, and Czechia occupied the top five, while Finland ranked sixth; the rank correlation with the main ranking among the common countries was 0.963. The widening of intervals in the middle ranks demonstrates the need to avoid interpreting small rank differences as a definitive indicator of superiority (Table 3). Detailed bootstrap results are provided in the Supplementary Materials. These bootstrap rank intervals are visualized in Figure 2.

### 3.4. Sensitivity and Robustness

The broad country structure was preserved across most prespecified alternative specifications, but top-five membership was not invariant. Using domain weights of 60/40, using only the Q1 level instead of the Q1–Q5 difference, geometric aggregation, exclusion of pandemic years, and omission of several individual disease indicators retained the baseline top-five group. In contrast, the 40/60 weighting, equal weighting of all five indicators, the minimum-domain score, the 2016–2023 specification, omission of COPD, z-score normalization, and the balanced 18-country analysis changed at least one top-five member. Table 4 therefore reports both rank correlations and the corresponding top-five membership and maximum rank change. Finland was the least stable member of the baseline top five: it left the top five in seven of the sixteen specifications reported in Table 4, whereas the Netherlands and Slovenia remained in the top five throughout, and Spain and Sweden were displaced only under equal weighting of all five indicators.

### 3.5. Spending-Adjusted Results

**Figure 3:**
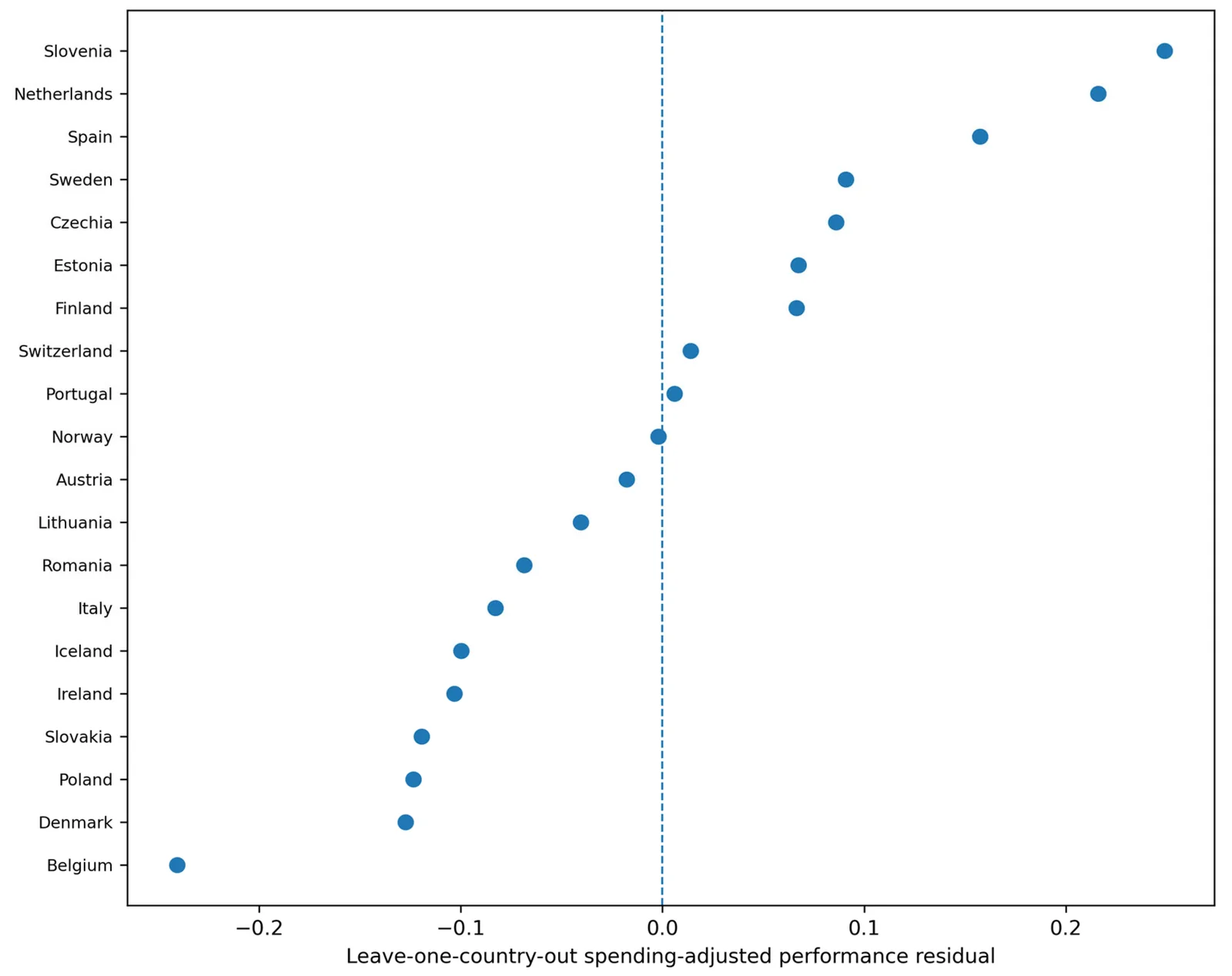
Spending-adjusted performance. Note. Expected performance for each country is estimated from a quadratic spending model fitted after excluding that country. Values above zero indicate above-predicted performance and values below zero indicate below-predicted performance. Source: Authors’ calculations.

**Figure 4:**
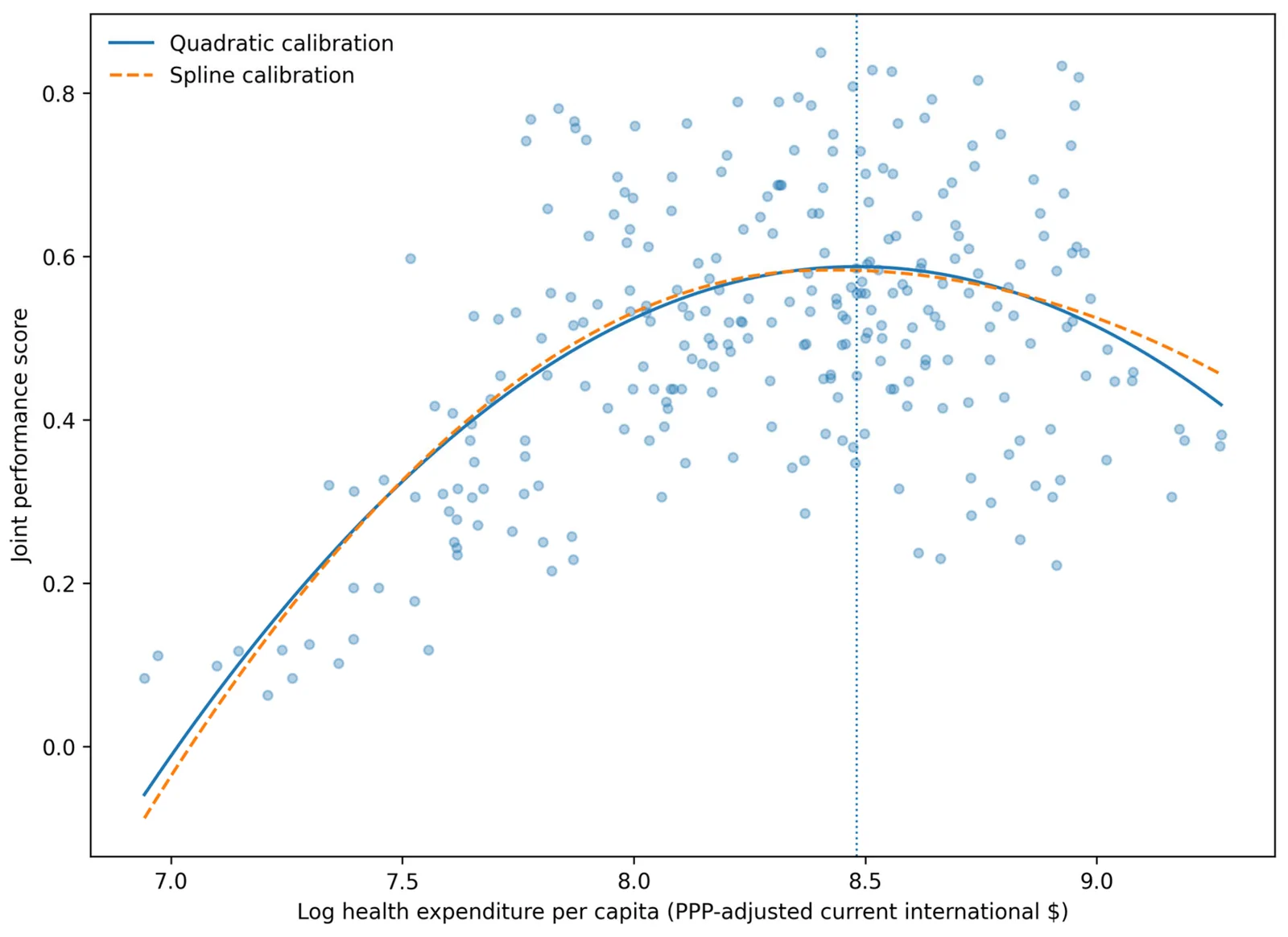
Joint performance and health expenditure calibration. Note. Points are country-year observations. Quadratic and spline curves are displayed at the 2016 year fixed effect. The vertical dotted line indicates the quadratic turning point (approximately 4826 PPP-adjusted current international dollars). The turning point is descriptive and is not interpreted as an optimal spending level. Source: Authors’ calculations.

When using the revised joint score based on the prespecified 20-country comparator set, the Akaike information criterion (AIC) for linear spending calibration is approximately −217.64, for the quadratic model −283.97, and for the flexible spline model −282.67. Leave-one-country-out predictive diagnostics showed that the two nonlinear forms performed almost identically and both clearly outperformed the linear specification: RMSE was 0.148 and MAE was 0.119 for the quadratic model, 0.150 and 0.121 for the spline model, and 0.170 and 0.138 for the linear model. The spline-based spending-adjusted ranking was materially unchanged from the quadratic ranking (Spearman ρ = 0.991), with the same top five and a maximum rank change of two positions. In the primary quadratic model based on leave-one-country-out estimation, the top five spending-adjusted positions are Slovenia, the Netherlands, Spain, Sweden, and Czechia. The rank correlation with the main current-spending specification is 0.997 when using health expenditure lagged by one year, 0.982 when using a strict three-year trailing average based on three complete consecutive expenditure observations, and 0.995 when year fixed effects are removed. The correlation with the alternative measure based on the share of health expenditure in GDP is 0.839. The fitted quadratic turning point is approximately 4826 PPP-adjusted current international dollars within the observed expenditure range of approximately 1035–10,602; this is a descriptive feature of the calibration curve and should not be interpreted as an optimal spending level. Country-level spending-adjusted results are reported in Table 5 and shown in Figure 3; the spending–performance calibration is shown in Figure 4.

## 4. Discussion

This study examined European health systems by considering two areas that are often addressed separately in comparisons: income-related inequality in cost-related unmet medical care, and avoidable hospitalizations for primary-care-sensitive chronic conditions. The key finding is that the upper part of the ranking is relatively stable across many prespecified methodological variations, while top-five membership changes under some alternative weighting, disease-omission, scaling, and balanced-period specifications. This suggests that focusing on performance groups and domain profiles rather than single rank numbers is more accurate in country comparisons.

The fact that the Netherlands, Slovenia, and Spain remain in the top group in both the observed joint ranking and moving-block bootstrap analyses indicates that the results are not determined by a single year or a single indicator. Sweden and Finland are also in the baseline top group; however, the rank intervals widen when temporal dependence is considered with three-year blocks, and Finland moves to sixth place in the fixed 18-country balanced 2016–2023 analysis. This pattern is consistent with broader cautions in composite-indicator research against over-interpreting small differences in rank [19]. Across the sixteen sensitivity specifications in Table 4, Finland left the top five in seven, whereas the Netherlands and Slovenia remained in the top five throughout and Spain and Sweden left it only under equal weighting of all five indicators. Taken together, four of the five baseline upper positions were comparatively robust, whereas Finland’s fifth position was more specification-sensitive; Finland also ranked seventh in the spending-adjusted analysis.

The distinction between the two domains is an important aspect of the study. Income-related inequality in cost-related unmet care corresponds to a different policy question than chronic-disease hospitalization. A country’s relatively low hospitalization rates do not necessarily mean that financial barriers are limited for low-income groups. Similarly, a small cost-related gap between income groups does not, by itself, indicate effective outpatient management of chronic diseases. The OECD’s current performance framework, which treats equity as a dimension that cuts across other dimensions, supports this distinction [4].

The main limitation regarding the domain of unmet medical needs stems from the measurement structure. The traditional EU-SILC rate uses the entire sample as the denominator instead of those who need healthcare, thus combining healthcare need with failure to obtain care [12]. Therefore, the Q1–Q5 difference in our study was not termed “access equity.” However, monitoring the cost-related gap between income groups for unmet needs is still important for the healthcare system because it makes visible social groups that fall below the national average. Using subgroup-specific indicators to monitor health inequality is one of the key approaches recommended by the WHO [7]. The Q1–Q5 gap also uses only the two ends of the income distribution and therefore does not describe the full socioeconomic gradient; a small gap may occur at either low or high overall levels of unmet need.

The 50/50 weighting of the composite score is not an objective weighting determined by the data. This choice is based on the assumption that income-related inequality and primary-care-sensitive hospitalization outcomes have equal conceptual importance in the research question. Sensitivity results show that this choice alone does not determine the country ranking. The high correlation at 40/60 and 60/40 weights, the geometric mean agreement at 0.946, and the largely preserved ranking when diseases are removed individually are reassuring in this respect. In contrast, the fact is that the correlation drops to 0.770 under the non-compensatory minimum-domain measure, and Finland is replaced by Norway in the top five; thus, sensitivity to the aggregation rule extends beyond the middle ranks. This is not a weakness to be hidden; it is a result of normative decisions inherent in composite indicators that should be explicitly reported [19]. The largest reordering occurred under equal weighting of all five indicators (ρ = 0.692), which effectively assigns 80% of the composite weight to the hospitalization domain.

The primary purpose of spending adjustment is not to declare countries “efficient” or “inefficient.” The concept of technical efficiency requires the production relationship of numerous inputs and outputs; a single expenditure variable is insufficient to establish this relationship [25]. The leave-one-country-out estimate used in the study answers a more limited question: how much is a country’s joint performance above or below the performance level predicted by health expenditure and year conditions observed in other countries? Slovenia’s rise to first place in this measure shows that observed joint performance and performance relative to resource intensity are not the same concept. Because the fitted quadratic relationship is non-monotonic within the observed spending range, the residuals should be interpreted only as deviations from the empirical calibration curve and not as evidence that additional spending reduces performance beyond the turning point.

This non-monotonic calibration also affects how individual movements in Table 5 should be read. The fitted turning point is approximately 4826 international dollars, close to the sample mean of 4407, so the observed data span both the ascending and descending portions of the calibration curve. Consequently, movements such as Romania from 20th to 13th and Estonia from 11th to 6th, compared with Denmark from 15th to 19th and Belgium from 17th to 20th, reflect position relative to this empirical curve rather than a monotonic return to spending. These residual-based movements should therefore not be interpreted as measures of value for money or technical efficiency.

The most useful aspect of the study from a policy standpoint is not so much its precise ranking, but rather its demonstration of the extent to which countries diverge. A country with a relatively weak cost-related inequality score and a strong hospitalization score might prioritize financial barriers and access to services for low-income groups. A country with the opposite profile, while maintaining its relative advantage in access, might focus on chronic disease monitoring, continuity of care, or the organization of hospital use. This comparison cannot determine which policy produced the result; however, it can indicate which areas should be examined for detailed country studies. Several limitations should be noted. The EU-SILC indicator is self-reported and, because it takes the entire population as a denominator, it cannot fully separate health need from barriers to access. Although avoidable hospitalizations are sensitive to primary care, they are not specific to primary care. Ranking-based scores show position relative to the comparison set of the same year, not absolute improvement. The requirement of at least ten years of data increases comparability but limits the sample to 20 countries. The moving-block bootstrap accounts for temporal variability but does not propagate all sampling and measurement errors in the source datasets. Finally, the analysis is country-level; it cannot be concluded that individuals who cannot receive care due to cost and those who experience avoidable hospitalizations are the same individuals. The two domains also refer to slightly different age thresholds because the source datasets use 16 years and over for EU-SILC unmet need and 15 years and over for OECD admissions. Rank normalization discards information on the absolute distance between countries and makes scores dependent on the contemporaneous comparator set.

## 5. Conclusions

In the baseline joint ranking, the Netherlands, Slovenia, Spain, Sweden, and Finland occupied the top five, although Finland’s position was notably more sensitive to alternative specifications than those of the other four countries. The two domains also revealed distinct country profiles, indicating that relatively favorable cost-related inequality performance does not necessarily coincide with favorable primary-care-sensitive hospitalization performance. Spending adjustment changed several country positions and placed Slovenia, the Netherlands, Spain, Sweden, and Czechia in the top five, reinforcing that observed joint performance and performance relative to the empirical spending calibration are different concepts.

The main methodological recommendation of the study is to make normative preferences visible in healthcare system comparisons, to keep indicators of inequality alongside mean results, and to report ranking uncertainty as part of the results. The value of health system comparisons lies not in producing a definitive “best country” ranking, but in showing which countries are strong or fragile in which dimensions and to what extent this finding remains robust to methodological choices.

## Figures and Tables

**Table 1:** Key indicators used in the study.

Domain	Indicator	Definition	Unit	Source	Favourable Direction
Cost-related inequality	Q1 unmet need	Cost-related unmet medical care, lowest income quintile	%	Eurostat, hlth_silc_08	Lower
Cost-related inequality	Q5 unmet need	Cost-related unmet medical care, highest income quintile	%	Eurostat, hlth_silc_08	Lower
Cost-related inequality	Q1–Q5 gap	Absolute difference between Q1 and Q5	Percentage points	Authors’ calculation	Lower
Hospitalization	Asthma	Avoidable asthma admission	Per 100,000	OECD HCQO	Lower
Hospitalization	COPD	Avoidable COPD admission	Per 100,000	OECD HCQO	Lower
Hospitalization	CHF	Avoidable congestive heart failure admission	Per 100,000	OECD HCQO	Lower
Hospitalization	Diabetes	Avoidable diabetes admission	Per 100,000	OECD HCQO	Lower
Resource	Health expenditure	Current health expenditure per capita, PPP-adjusted	Current international $	World Bank/WHO GHED	—

Note. OECD admission indicators are age- and sex-standardized rates for the population aged 15 years and over. Because the EU-SILC unmet-need indicator uses the full valid population as the denominator, the Q1–Q5 difference is not interpreted as a pure access probability.

**Table 2:** Descriptive statistics for the main sample.

Variable	N	Mean	SD	Median	Minimum	Maximum
Q1 cost-related unmet need (%)	275	2.15	2.72	1.00	0.00	14.50
Q5 cost-related unmet need (%)	275	0.26	0.48	0.10	0.00	4.30
Q1–Q5 gap (percentage points)	275	1.89	2.38	0.90	0.00	13.30
Asthma admissions	275	34.52	20.20	31.60	4.80	113.80
COPD admissions	275	175.55	89.46	158.20	18.10	453.70
CHF admissions	275	268.92	124.58	222.80	81.70	764.20
Diabetes admissions	275	124.69	71.15	107.10	30.70	357.00
Health expenditure per capita (PPP)	275	4406.80	1940.46	4312.06	1035.37	10,601.93

Note. Asthma, COPD, CHF and diabetes are admission rates per 100,000 population. Health expenditure is reported in PPP-adjusted current international dollars.

**Table 3:** Joint performance ranking and moving-block bootstrap uncertainty.

Country	Observed Years	Joint Score	Rank	95% Rank Interval	P (Top 5)	Spending-Adjusted Rank
Netherlands	14	0.748	1	1–4	100.00%	2
Slovenia	15	0.73	2	1–3	100.00%	1
Spain	15	0.68	3	1–4	99.90%	3
Sweden	15	0.636	4	3–7	90.67%	4
Finland	15	0.621	5	3–6	95.66%	7
Czechia	15	0.556	6	5–11	4.34%	5
Austria	15	0.535	7	6–11	0.99%	11
Norway	15	0.529	8	5–11	5.09%	10
Portugal	12	0.523	9	7–10	0.00%	9
Switzerland	15	0.516	10	5–13	3.16%	8
Estonia	10	0.509	11	7–14	0.19%	6
Iceland	12	0.469	12	11–14	0.00%	15
Italy	14	0.468	13	9–15	0.00%	14
Ireland	15	0.458	14	12–15	0.00%	16
Denmark	15	0.441	15	10–16	0.00%	19
Lithuania	12	0.374	16	15–18	0.00%	12
Belgium	14	0.332	17	16–19	0.00%	20
Slovakia	12	0.317	18	17–18	0.00%	17
Poland	15	0.22	19	18–20	0.00%	18
Romania	10	0.173	20	19–20	0.00%	13

Note. Rank intervals are the integer bounds derived from the 2.5th and 97.5th percentiles of the valid three-year synchronized moving-block bootstrap rank distributions. The 2.5th percentile was rounded downward and the 97.5th percentile upward. Valid replications ranged from 9995 to 10,000 across countries. These are temporal-resampling rank intervals, not conventional sampling-based confidence intervals. The spending-adjusted rank is derived from the leave-one-country-out nonlinear spending equation.

**Table 4:** Selected sensitivity analyses of the main ranking.

Sensitivity Analysis	Spearman ρ	N	Top Five	Max Δrank
40% inequality/60% hospitalization weights	0.898	20	NLD, SVN, ESP, SWE, PRT	6
60% inequality/40% hospitalization weights	0.943	20	NLD, SVN, ESP, FIN, SWE	5
Q1 level instead of Q1–Q5	0.997	20	NLD, SVN, ESP, SWE, FIN	1
Equal weighting of all five indicators	0.692	20	PRT, ITA, NLD, ISL, SVN	11
Geometric mean of the two domains	0.946	20	NLD, SVN, ESP, SWE, FIN	6
Non-compensatory minimum-domain score	0.770	20	NLD, SVN, ESP, SWE, NOR	7
Exclude 2020–2021	0.989	20	NLD, SVN, ESP, FIN, SWE	2
2015–2023	0.968	19	NLD, ESP, SVN, SWE, FIN	4
2016–2023	0.961	19	ESP, NLD, SWE, SVN, CZE	4
Omit asthma	0.958	20	NLD, SVN, ESP, FIN, SWE	5
Omit COPD	0.931	20	NLD, ESP, SVN, SWE, NOR	5
Omit CHF	0.958	20	SVN, NLD, SWE, ESP, FIN	4
Omit diabetes	0.982	20	SVN, NLD, SWE, ESP, FIN	3
Within-year z-score scaling	0.923	20	NLD, SVN, ESP, SWE, CHE	5
Fixed 18-country balanced panel, 2016–2023	0.963	18	ESP, SWE, NLD, SVN, CZE	3
Minimum leave-one-year-out rank correlation	0.991	20	NLD, SVN, ESP, SWE, FIN	2

Note. ρ = 1 indicates complete rank agreement and ρ = 0 no rank association. Country codes in the top-five column use ISO3 abbreviations. The equal-five-indicator alternative assigns 80% of the total weight to the hospitalization domain and is therefore conceptually different from the main two-domain design.

**Table 5:** Spending-adjusted country performance.

Country	LOCO Spending-Adjusted Residual	Adjusted Rank	Main Joint Rank
Slovenia	0.249	1	2
Netherlands	0.216	2	1
Spain	0.158	3	3
Sweden	0.091	4	4
Czechia	0.086	5	6
Estonia	0.067	6	11
Finland	0.066	7	5
Switzerland	0.014	8	10
Portugal	0.006	9	9
Norway	−0.002	10	8
Austria	−0.018	11	7
Lithuania	−0.041	12	16
Romania	−0.068	13	20
Italy	−0.083	14	13
Iceland	−0.100	15	12
Ireland	−0.103	16	14
Slovakia	−0.119	17	18
Poland	−0.124	18	19
Denmark	−0.127	19	15
Belgium	−0.241	20	17

Note. A positive residual indicates joint performance above that predicted from the nonlinear spending–performance relationship estimated on the other countries. This residual is not a measure of technical efficiency.

## Data Availability

The processed country-year dataset with country names and ISO3 codes, the full expenditure series used to construct strict lagged and three-year measures, and the replication code are provided in Supplementary Materials. The underlying indicators are publicly available from Eurostat (hlth_silc_08), OECD (DSD_HCQO@DF_PC), and the World Bank (SH.XPD.CHEX.PP.CD). The OECD admission series were retrieved from the SDMX dataflow OECD.ELS.HD, DSD_HCQO@DF_PC, version 2.1, with the query beginning in 2000. The replication materials are also archived in https://zenodo.org/records/23047353.

## Supplementary Materials

The following supporting information can be downloaded at: https://www.mdpi.com/article/10.3390/healthcare14193294/s1; Table S1: Core-sample coverage and valid three-year bootstrap replications; Table S2: Two-year and three-year moving-block bootstrap results; Table S3: Balanced 18-country 2016–2023 ranking and bootstrap; Table S4: Headline changes across sensitivity specifications; Table S5: Spending-model diagnostics; Table S6: Broad contextual characteristics of the 20 included health systems.
