# Supplementary material for: Equity Meets Primary-Care-Sensitive Performance: A Longitudinal Benchmark of Cost-Related Unmet Care and Avoidable Hospitalization in Europe
Source: Healthcare (Basel). 2026 Oct 2;14(19):3294. doi: 10.3390/healthcare14193294 (PMC13664954; doi:10.3390/healthcare14193294)
Supplement: Supplementary file 1 [file healthcare-14-03294-s001.zip › healthcare-4585896-supplementary.pdf]

Supplementary Materials

Manuscript ID: healthcare-4585896

Equity Meets Primary-Care-Sensitive Performance: A Longitudinal Benchmark of Cost-Related Unmet Care and Avoidable Hospitalization in Europe

Table S1. Core-sample coverage and valid three-year bootstrap replications

Country	ISO3	Observed years	Valid 3-year MBB replications
Austria	AUT	15	10000
Belgium	BEL	14	10000
Switzerland	CHE	15	10000
Czechia	CZE	15	10000
Denmark	DNK	15	10000
Spain	ESP	15	10000
Estonia	EST	10	9995
Finland	FIN	15	10000
Ireland	IRL	15	10000
Iceland	ISL	12	10000
Italy	ITA	14	10000
Lithuania	LTU	12	10000
Netherlands	NLD	14	10000
Norway	NOR	15	10000
Poland	POL	15	10000
Portugal	PRT	12	10000
Romania	ROU	10	9995
Slovakia	SVK	12	10000
Slovenia	SVN	15	10000
Sweden	SWE	15	10000

Table S2. Two-year and three-year moving-block bootstrap results

Country	3-year rank interval	3-year P(top 5)	2-year rank interval	2-year P(top 5)
Austria	6–11	0.99%	6–13	0.48%
Belgium	16–19	0.00%	16–18	0.00%
Switzerland	5–13	3.16%	6–13	2.03%
Czechia	5–11	4.34%	5–10	2.80%
Denmark	10–16	0.00%	11–16	0.00%
Spain	1–4	99.90%	1–4	99.95%
Estonia	7–14	0.19%	7–14	0.12%
Finland	3–6	95.66%	3–6	97.19%
Ireland	12–15	0.00%	11–15	0.00%
Iceland	11–14	0.00%	11–14	0.00%
Italy	9–15	0.00%	10–15	0.00%
Lithuania	15–18	0.00%	15–17	0.00%
Netherlands	1–4	100.00%	1–3	100.00%
Norway	5–11	5.09%	6–12	2.13%
Poland	18–20	0.00%	19–20	0.00%
Portugal	7–10	0.00%	7–10	0.00%
Romania	19–20	0.00%	19–20	0.00%
Slovakia	17–18	0.00%	17–18	0.00%
Slovenia	1–3	100.00%	1–3	100.00%
Sweden	3–7	90.67%	3–6	95.29%

Table S3. Balanced 18-country 2016–2023 ranking and bootstrap

Country	Mean score	Rank	95% bootstrap rank interval	P(top 5)
Spain	0.742	1	1–4	100.00%
Sweden	0.739	2	1–4	100.00%
Netherlands	0.739	3	1–4	100.00%
Slovenia	0.730	4	2–4	100.00%
Czechia	0.619	5	5–6	67.03%
Finland	0.612	6	5–6	32.97%
Austria	0.562	7	7–9	0.00%
Portugal	0.533	8	8–10	0.00%
Estonia	0.508	9	7–11	0.00%
Norway	0.476	10	7–12	0.00%
Switzerland	0.466	11	8–13	0.00%
Ireland	0.427	12	10–13	0.00%
Lithuania	0.392	13	11–15	0.00%
Denmark	0.375	14	12–16	0.00%
Slovakia	0.318	15	14–17	0.00%
Poland	0.282	16	14–17	0.00%
Belgium	0.280	17	16–18	0.00%
Romania	0.199	18	17–18	0.00%

Table S4. Headline changes across sensitivity specifications

Specification	Spearman ϱ	Countries	Top-five countries	Maximum rank change	Top-five membership change
40/60	0.898	20	NLD; SVN; ESP; SWE; PRT	6	FIN, PRT
60/40	0.943	20	NLD; SVN; ESP; FIN; SWE	5	—
Q1 instead gap	0.997	20	NLD; SVN; ESP; SWE; FIN	1	—
equal five	0.692	20	PRT; ITA; NLD; ISL; SVN	11	ESP, FIN, ISL, ITA, PRT, SWE
geometric	0.946	20	NLD; SVN; ESP; SWE; FIN	6	—
minimum-domain	0.770	20	NLD; SVN; ESP; SWE; NOR	7	FIN, NOR
exclude 2020-2021	0.989	20	NLD; SVN; ESP; FIN; SWE	2	—
2015-2023	0.968	19	NLD; ESP; SVN; SWE; FIN	4	—
2016-2023	0.961	19	ESP; NLD; SWE; SVN; CZE	4	CZE, FIN
omit asthma	0.958	20	NLD; SVN; ESP; FIN; SWE	5	—
omit copd	0.931	20	NLD; ESP; SVN; SWE; NOR	5	FIN, NOR
omit chf	0.958	20	SVN; NLD; SWE; ESP; FIN	4	—
omit diabetes	0.982	20	SVN; NLD; SWE; ESP; FIN	3	—
z-score	0.923	20	NLD; SVN; ESP; SWE; CHE	5	CHE, FIN
balanced 18 2016-2023	0.963	18	ESP; SWE; NLD; SVN; CZE	3	CZE, FIN
leave-one-year-out min	0.991	20	NLD; SVN; ESP; SWE; FIN	2	varies by omitted year

Table S5. Spending-model diagnostics

Model	AIC	LOCO RMSE	LOCO MAE
linear	-217.64	0.170	0.138
quadratic	-283.97	0.148	0.119
spline	-282.67	0.150	0.121

Quadratic turning point: 4826 PPP-adjusted current international dollars. Observed expenditure range: 1035–10602. The turning point is a descriptive property of the fitted curve and is not interpreted as an optimal spending level.

Comparison	Spearman ρ
current_vs_lag1	0.997
current_vs_strict_trailing3	0.982
current_vs_gdp_share	0.839
current_vs_no_year_fe	0.995

Table S6. Broad contextual characteristics of the 20 included health systems

Country	Broad financing/coverage arrangement	Primary-care pathway / gatekeeping context
Austria	Compulsory social health insurance plus general taxation; responsibilities split across federal, Länder, and insurance actors	No formal gatekeeping; patients can directly access GPs, specialists, and hospitals.
Belgium	Compulsory social health insurance with federal and federated responsibilities	No formal gatekeeping; direct specialist access and free provider choice.
Switzerland	Mandatory health insurance with competing insurers and substantial cantonal responsibilities	Direct access under traditional insurance; gatekeeping applies in managed-care insurance options.
Czechia	Compulsory social health insurance with multiple quasi-public insurance funds	No true gatekeeping; direct access to specialists is common.
Denmark	Predominantly tax-funded, regionally and municipally organized universal system	GPs are central first-contact providers and strong gatekeepers to specialist and hospital care.
Spain	Tax-funded, decentralized National Health System with regional delivery	Family doctors are first contact and gatekeepers to specialist care; emergency care can be accessed directly.
Estonia	Centralized social health insurance managed by the Estonian Health Insurance Fund	Family physicians have a partial gatekeeping role; referral is required for most specialists, with defined exceptions.
Finland	State-financed regional public system, complemented by national health insurance and occupational-care channels	The public pathway has strong primary-care gatekeeping to specialist services.
Ireland	Primarily tax-funded mixed public-private system	GPs are usual first-contact providers, but entitlement and charges vary; universal free primary care is not available.
Iceland	State-centred, publicly funded universal system	Primary care is formally the first contact, but direct access to medical specialists remains common.
Italy	Tax-funded, regionally organized National Health Service	Residents register with a GP/paediatrician; GPs act as gatekeepers and care coordinators.
Lithuania	Compulsory social health insurance through the National Health Insurance Fund plus state transfers	Primary care is the routine first contact and has a gatekeeping function, with some direct-access exceptions.
Netherlands	Regulated social insurance for curative care with competing private insurers	Strict GP gatekeeping to hospital and specialist care.
Norway	Predominantly publicly financed universal system; municipalities organize primary care and regions organize specialist care	List-based regular-GP system; GPs act as gatekeepers to specialist care.
Poland	Social health insurance with substantial territorial-government roles in service organization	Primary care is the entry point and serves as gatekeeper to more specialized care.

Country	Broad financing/coverage arrangement	Primary-care pathway / gatekeeping context
Portugal	Predominantly tax-funded universal National Health Service, alongside occupational health subsystems	GPs act as gatekeepers to specialist and secondary care.
Romania	Social health insurance supplemented by state and local budgets	Family physicians have a gatekeeping role, with direct specialist access for specified conditions.
Slovakia	Compulsory social health insurance with three competing insurers	GP gatekeeping is formal but weakly enforced; patients often access specialists directly.
Slovenia	Single-purchaser compulsory social health insurance with a relatively centralized system	GPs act as gatekeepers to specialized care.
Sweden	Predominantly tax-funded, regionally organized universal system	No formal gatekeeping in most regions; direct specialist access is generally possible.

Note. These are intentionally broad contextual characterizations and are not analytic covariates in the benchmark. Source: European Observatory on Health Systems and Policies, country overviews and Health Systems and Policy Monitor (HSPM), accessed 28 September 2026.
